# A Stoma’s Stowaway: A Rare Case of Parastomal Herniation of the Gallbladder

**DOI:** 10.7759/cureus.97642

**Published:** 2025-11-24

**Authors:** Philip Macanovic, Zachary James-Knights, Ame Saidy, Tarek Elshafey, Muhammad Effendi

**Affiliations:** 1 General Surgery, York and Scarborough Teaching Hospitals NHS Foundation Trust, York, GBR

**Keywords:** computed tomography, endoscopic retrograde cholangiopancreatography, gallbladder, gallstone pancreatitis, ileostomy, pancreatitis, parastomal hernia

## Abstract

Parastomal herniation (PSH) of visceral organs represents an exceptionally uncommon phenomenon, most often encountered in surgically complex, multimorbid patients. These cases often present significant diagnostic and surgical challenges. Involvement of the gallbladder is particularly uncommon but clinically important, due to the potential risk of complications such as cholecystitis, torsion, and perforation. We describe a case of gallbladder-containing PSH in a 76-year-old female with an extensive surgical history and ileostomy in situ. The hernia was initially managed with manual reduction, which successfully restored the gallbladder to its anatomical position. Subsequently, the patient developed gallstone pancreatitis, necessitating endoscopic retrograde cholangiopancreatography (ERCP).

This report underscores the clinical uncertainty surrounding the choice between conservative and operative strategies in this high-risk cohort. Although conservative approaches may be feasible in select cases, the long-term durability of non-operative management and the risk of recurrence or delayed biliary complications remain unclear. Currently, there is no consensus on the optimal surgical management of these presentations, and decisions are guided by individual case characteristics. This report emphasises the importance of early imaging, vigilant monitoring, and a multidisciplinary approach to optimise patient outcomes.

## Introduction

Stoma formation is a frequent outcome of surgical intervention for intestinal pathology, including colorectal cancer, ischaemic colitis, and inflammatory bowel disease. In the UK, over 100,000 individuals live with stomas, with ileostomies comprising approximately 39% [1]. Although procedurally simple, stomas are often associated with significant long-term morbidity, with patient-related complications such as leakage and soiling, alongside surgical complications including stoma stenosis, retraction, and parastomal herniation (PSH) [2]. PSH is characterised as an abnormal protrusion of intra-abdominal contents through an abdominal wall defect created during the formation of a stoma and may typically only contain loops of small bowel [3]. Reported incidence is variable on account of heterogeneity in definition, diagnostic modalities, follow-up duration, and type of stoma [4]. Despite this variability, PSH is a common postoperative complication, affecting up to 50% of patients and contributing substantially to morbidity.

We present a rare case of PSH containing a gallbladder in a patient with a significant previous surgical history. We highlight the necessity of careful consideration to ensure that management strategies are appropriately tailored to the patient’s morbidity profile. Additionally, we emphasise the importance of exercising clinical judgment when opting for non-operative management, given the potential for recurrence and delayed biliary complications.

## Case presentation

A 76-year-old female presented with a two-day history of generalised abdominal pain associated with nausea and non-bilious vomiting. She had an extensive surgical history, including a previous total abdominal hysterectomy and sigmoid colectomy, complicated by recurrent small bowel obstruction, which had later required ileostomy formation. She was an ex-smoker with a medical history of chronic obstructive pulmonary disease (COPD) and asthma.

At the time of admission, her blood pressure was 123/72 mmHg, heart rate was 77 beats per minute, respiratory rate was 22 breaths per minute, and oxygen saturation was 92% on room air. She was apyrexial. Physical examination identified an active, right-sided ileostomy with a soft, reducible parastomal hernia. Palpation revealed generalised abdominal tenderness, most pronounced in the right upper quadrant and around the ileostomy site. Admission serological workup identified the following: white blood cell count 18.7 x10^9^/L, neutrophils 13.92 x10^9^/L, C-reactive protein (CRP) 12 mg/L, alkaline phosphatase (ALP) 93 IU/L, alanine aminotransferase 6 IU/L, bilirubin 8 µmol/L, lactate 1.2 mmol/L, and amylase 49 U/L. Kidney function, urea, and electrolytes were unremarkable. She was subsequently commenced on locally guided empirical antibiotics for suspected intra-abdominal infection, including tigecycline, levofloxacin, and gentamicin.

**Figure 1:**
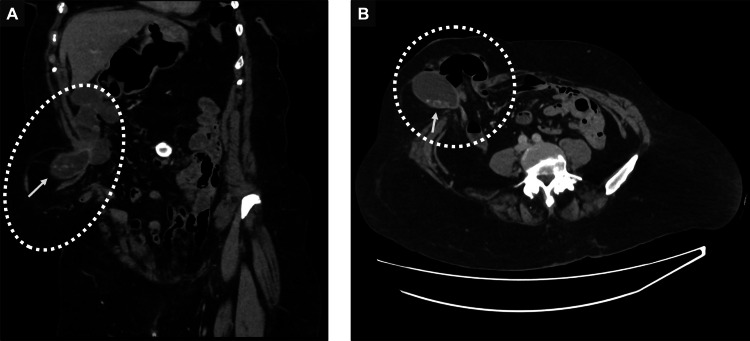
CT abdomen Contrast-enhanced CT abdomen imaging in sagittal (A) and axial (B) views, demonstrating a right-sided parastomal hernia (dotted circle) containing gallbladder with intraluminal gallstones (arrows) and adjacent small bowel loops with mild upstream partial obstruction CT: computed tomography

Mid-stream urinalysis and chest X-ray were unremarkable. A subsequent contrast-enhanced CT scan of the abdomen revealed a parastomal hernia containing the gallbladder with gallstones as well as small-bowel loops. Only minimal upstream small-bowel dilatation (up to 23 mm) and mild biliary duct dilatation tapering distally were noted, with no evidence of an obstructing lesion, cholecystitis, or pancreatitis (Figure 1). The general surgery team assessed her as unsuitable for operative intervention, manually reduced the hernia, and opted to continue conservative management with intravenous antibiotics.

Five days later, the patient developed acute severe epigastric pain with tachycardia at 114 beats per minute and reduced oxygen saturation to 88% on room air. Repeat blood tests identified an increased amylase of 510 U/L, white blood cell count of 28.5 x10^9^/L, neutrophils of 23.91 x10^9^/L, and CRP of 277 mg/L. Lactate, electrolytes, and liver function tests were unremarkable. The preceding CT abdomen scan confirmed that the gallbladder had returned to its anatomical position with new pancreatic swelling and peri-pancreatic oedematous fat stranding suggestive of acute pancreatitis. Supportive management was initiated, including analgesia, controlled supplementary oxygen, and intravenous fluids.

Further evaluation with magnetic resonance cholangiopancreatography (MRCP) revealed several distal gallstones within the common bile duct, measuring up to 7 mm, including a stone at the ampulla. There was no intra or extra-hepatic biliary duct or pancreatic duct dilatation. Incidentally, variant biliary anatomy was noted, with aberrant insertion of the right posterior hepatic duct into the common hepatic duct.

**Figure 2:**
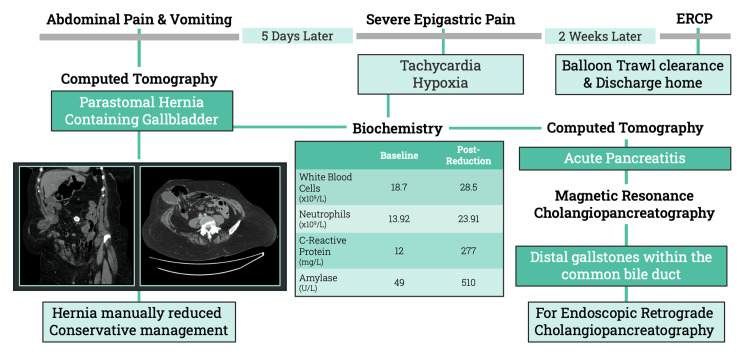
Timeline of clinical events A timeline summarising the key imaging, biochemical trends, and interventions to illustrate decision-making surrounding non-operative management of parastomal hernia containing gallstones, and subsequent biliary complications ERCP: endoscopic retrograde cholangiopancreatography

Two weeks later, following the resolution of pancreatitis-related symptoms and inflammation, endoscopic retrograde cholangiopancreatography (ERCP) was performed, with successful sphincterotomy and balloon trawl clearance of five ductal stones, the largest measuring 8 mm. Post-procedure, she remained asymptomatic and was discharged home the following day. A timeline of clinical and biochemical events is outlined in Figure 2.

## Discussion

This report highlights the judicious use of conservative management in a rare gallbladder-containing parastomal hernia (gPSH). To our knowledge, only 18 cases [5] of gPSH have been reported to date. Gallbladder migration into a parastomal defect is a peculiar phenomenon that may arise within a unique biomechanical environment. As with our case, gPSH shows marked female predominance in up to 90% of cases, with a mean age of 77 at presentation [6]. These cohorts are thought to have diminished visceral fat, resulting in the elongation and laxity of the mesentery, a recognised risk factor for gallbladder torsion [7]. In combination with repeated increased intra-abdominal pressure in COPD and local adhesions from abdominal surgery, this may facilitate the displacement of a mobile gallbladder into the parastomal defect.

There is no clear surgical consensus on how best to manage these cases, underscoring the importance of a holistic assessment of each patient’s operative suitability. Surgical intervention typically consists of cholecystectomy, often with synchronous hernia repair, and is favoured in the majority of cases [6]. This rare anatomical variation presents unique considerations for operative access. Previous reports have described successful cholecystectomy and repair performed entirely through the parastomal opening [8], and more recently, a case of open cholecystostomy via the parastomal hernia under local anaesthesia [5].

In the present case, conservative management was considered most appropriate given the patient’s clinical factors and operative risk. A non-operative approach with a known mobile gallbladder carries the risk of serious sequelae, including gallbladder torsion, necrosis, and perforation [6]. Clinicians should maintain a high index of suspicion for torsion of the gallbladder. Additionally, some authors have cautioned that attempts to reduce the hernia in such scenarios may trigger rupture of a fragile, gangrenous gallbladder, resulting in intra-abdominal contamination [9].

Our patient did not present with complicating features suggestive of acute cholecystitis or torsion and was therefore amenable to conservative management. We speculate that manual reduction may contribute to the displacement of intra-cholecystic gallstones into the common bile duct. MRCP-confirmed choledocholithiasis and a new rise in amylase support a plausible mechanism whereby manipulation of the gallbladder may have transiently straightened the cystic duct, allowing small calculi to pass and impact at the ampulla with subsequent development of acute pancreatitis requiring ERCP. This suggests the need for close monitoring following a reduction in similar cases, including serial liver enzymes alongside a low threshold for early ductal imaging if clinical or biochemical features of biliary pathology arise.

## Conclusions

Gallbladder involvement in a parastomal hernia is a rare yet clinically significant complication of stoma formation that is most commonly encountered in elderly, surgically complex, multimorbid patients. Whilst there is no current consensus on management, decisions should be individualised to each patient with careful consideration of patient health and the relative risks of operative and non-operative approaches, respectively. We demonstrate that conservative reduction can be a pragmatic approach in high-risk patients but may precipitate biliary complications. This report highlights the importance of early imaging, vigilant monitoring, and a multidisciplinary approach to optimise patient outcomes in these cohorts.

## References

[1] Black P (2009). Stoma care nursing management: cost implications in community care. Br J Community Nurs.

[2] Robertson I, Leung E, Hughes D, Spiers M, Donnelly L, Mackenzie I, Macdonald A (2005). Prospective analysis of stoma-related complications. Colorectal Dis.

[3] Śmietański M, Szczepkowski M, Alexandre JA (2014). European Hernia Society classification of parastomal hernias. Hernia.

[4] (2018). Prevention and treatment of parastomal hernia: a position statement on behalf of the Association of Coloproctology of Great Britain and Ireland. Colorectal Dis.

[5] Bakmiwewa SM, Vaska A, Idrees M, Farooque Y (2025). Gallbladder in the wrong neighbourhood: tackling para-urostomal herniated cholecystitis with open cholecystostomy. Cureus.

[6] Olusola S, Jarman T, Parmar C, Kathirvel M (2024). Parastomal gallbladder herniations: a systematic review. Cureus.

[7] Janakan G, Ayantunde AA, Hoque H (2008). Acute gallbladder torsion: an unexpected intraoperative finding. World J Emerg Surg.

[8] St. Peter SD, Heppell J (2005). Surgical images: soft tissue: incarcerated gallbladder in a parastomal hernia. Can J Surg.

[9] Benzoni C, Benini B, Pirozzi C (2004). Gallbladder strangulation within an incisional hernia. Hernia.

